# Genetic Instability and Intratumoral Heterogeneity in Neuroblastoma with *MYCN* Amplification Plus 11q Deletion

**DOI:** 10.1371/journal.pone.0053740

**Published:** 2013-01-14

**Authors:** Eva Villamón, Ana P. Berbegall, Marta Piqueras, Irene Tadeo, Victoria Castel, Anna Djos, Tommy Martinsson, Samuel Navarro, Rosa Noguera

**Affiliations:** 1 Department of Pathology, Medical School, University of Valencia, Valencia, Spain; 2 Research Foundation of Hospital Clínico Universitario of Valencia, Valencia, Spain; 3 Pediatric Oncology Unit, Hospital Universitario La Fe, Valencia, Spain; 4 Department of Clinical Genetics, The Sahlgrenska Academy, University of Gothenburg, Sahlgrenska University Hospital, Gothenburg, Sweden; Sapporo Medical University, Japan

## Abstract

**Background/Aim:**

Genetic analysis in neuroblastoma has identified the profound influence of *MYCN* amplification and 11q deletion in patients’ prognosis. These two features of high-risk neuroblastoma usually occur as mutually exclusive genetic markers, although in rare cases both are present in the same tumor. The purpose of this study was to characterize the genetic profile of these uncommon neuroblastomas harboring both these high-risk features.

**Methods:**

We selected 18 neuroblastomas with MNA plus 11q loss detected by FISH. Chromosomal aberrations were analyzed using Multiplex Ligation-dependent Probe Amplification and Single Nucleotide Polymorphism array techniques.

**Results and Conclusion:**

This group of tumors has approximately the same high frequency of aberrations as found earlier for 11q deleted tumors. In some cases, DNA instability generates genetic heterogeneity, and must be taken into account in routine genetic diagnosis.

## Introduction

Genetic instability and the presence of genetically heterogeneous cell populations are well-known features in neuroblastoma (NB), the most common extra-cranial solid neoplasm in childhood [1]–[5]. This disease is characterized by a diverse behavior, and both prognosis and response to therapy can vary widely [6]. Genetic markers of NB include *MYCN* amplification (MNA) and allelic loss of 11q. In addition, presence of gains and losses of heterozygosity (LOH) have been reported for other chromosomal regions [1], [7]. The use of Multiplex Ligation–dependent Probe Amplification (MLPA), Comparative Genomic Hybridization (CGH) and Single Nucleotide Polymorphisms arrays (aSNP) has provided accurate and rapid identification of genome abnormalities at high resolution [8]–[10]. In fact, numerical chromosome aberrations (NCAs), have been associated with excellent survival, while tumors with any type of segmental chromosome aberrations (SCAs) are related to a high-risk of relapse [1], [11].

MNA is present in about 17–20% of all NB cases, being associated with unfavorable cases with advanced stage disease, aggressive behavior and high-risk of relapse [6]. Intratumoral co-existence of amplified neuroblastic cells alongside non-amplified neuroblastic cells in the same tumor is regarded as heterogeneous MNA (hetMNA) [12]–[16] and presence of MNA in all neuroblastic cells is considered homogeneous MNA (homMNA) [13]. The study of heterogeneity according to the *MYCN* status requires single cell approach techniques such as fluorescence in situ hybridization (FISH). Several lines of investigation indicate that the q arm of chromosome 11 contains NB suppressor gene(s) [17]–[19]. Rearrangements in chromosome 11q occur also in approximately 30% of primary NB; they are associated with poor outcome and have recently been included as an independent risk factor in the International Neuroblastoma Risk Group (INRG) pretreatment risk classification [20]. An inverse relationship between MNA and 11q loss has been described, indicating distinct genetic subtypes of aggressive NB [19], [21]. In fact, infrequent cases with MNA plus 11q loss have been described with an unexpected complexity and dramatic decline of survival rates [22].

We carried out MLPA/aSNP studies of eighteen NB cases with MNA plus 11q loss detected by FISH, to characterize chromosomal aberrations and breakpoints and to describe the genetic cell heterogeneity in these unusual cases.

## Materials and Methods

### Tumor Material

Between 1999 and 2007, 905 tumor samples were referred to the Spanish Reference Centre for NB Biological and Pathological studies at the time of diagnosis. Nineteen samples were selected according to FISH status results for MNA and 11q deletion. Paraffin slides were stained with hematoxylin-eosin and examined by a pathologist (SN) to evaluate the amount of neuroblastic cells, and histopathologically categorized according to the International *Neuroblastoma* Pathology Classification (INPC) [23].

This study was approved by the Experimental Research Ethics Committee of the Spanish Society of Pediatric Hematology and Oncology (SHEOP) (File number: 59C18ABR2002; EC number: 2010-021396-81). Participants or their family members/informants signed written informed consent forms.

### FISH

*MYCN* copy number and integrity observed in chromosomal regions such as 1p36, 11q24 and 17q 22 were investigated in touch preparations and/or paraffin slides with commercial cocktail probes: *MYCN*(2p24)/*LAF*(2q11); *MLL*(11q23)/SE11; *MPO*(17q22)ISO17q/*p53*(17p13), (Kreatech, Biotechnology) and 1p36(*D1Z2*)/centromere Chromosome 1 (Qbiogene). Assessment and interpretation of FISH results were performed according to previously published procedures [24], [25].

### Ploidy

DNA content was analyzed by image cytometry following the protocol as previously described with minor changes, and the INRG recommendations [13], [26].

### Multilocus/Pangenomic Techniques

DNA from frozen tumors was extracted using phenol/chloroform/isoamyl alcohol extraction after proteinase K treatment. More than one tumor piece was analyzed in some hetMNA cases in order to extend the description of the tumor genotype. MLPA was used as a cost-effective first approach method in the detection of frequent SCAs in NB.

The technique was performed using the SALSA MLPA Kit P251/P252/P253 developed by MRC-Holland in co-operation with International Society of Paediatric Oncology European Neuroblastoma (SIOPEN). The SALSA MLPA P251 probemix contains probes for chromosomes 1, 3 and 11; P252 probemix for chromosomes 2 and 17; and P253 probemix for chromosomes 4, 7, 9, 12 and 14. Each panel includes control probes located in chromosome regions rarely altered numerically in NB. The technique and the interpretation guidelines are described elsewhere [8], [13].

For the array experiments GeneChip Human Mapping 250K arrays were used following the protocol provide by the supplier (Affymetrix, Inc., Santa Clara CA). The primary data analysis was made using GDAS software (Affymetrix), while genomic profiles were generated using CNAG (Copy Number Analyzer for Affymetrix GeneChip Mapping arrays) Version 3.0 with the AsCNAR (allele-specific copy-number analysis using anonymous references) function [27]. The UCSC genome browser, assembly February 2009 was used to visualize gene regions.

### Statistical Analysis

Overall survival (OS) was defined as the time to disease-related death or last follow-up. The clinical data were compared of 28 patients suffering from tumors with homogeneous MNA without 11q deletion (homMNA w/o 11q-del) tumors [28]. Survival curves were analyzed using the Kaplan-Meier method and compared using the log-rank test.

## Results

### Patients and Tumor Characteristics

Sample inclusion in this study was based solely on the co-existence of the MNA and 11q deletion aberrations detected by FISH in each sample. A total of nineteen primary tumors without previous treatment were selected for study using MLPA/aSNP. Clinical features of the patient cohort are summarized in Table 1. Patient age at time of diagnosis ranged from 9 to 108 months (median 24). Most of the patients (13/19) were older than 18 months. A slight predominance of males was present (63% versus 37%). The primary tumor was abdominal in all cases. All but two cases presented advanced stages, and thirteen patients had metastases at diagnosis. Bone and bone marrow metastases were the most frequent. Patients were treated according to Spanish Society of Paediatric Oncology (SEOP)/SIOPEN protocols depending on the date of diagnosis. Time to first relapse ranged from 4 to 28 months (mean 11.4 months). Mean follow-up was 32 months. Five patients remained alive 13 to 132 months after the initial diagnosis, 3/7 patients with hetMNA and 2/12 with homMNA (Table 2). Fourteen patients died, eleven due to disease progression and three because of sepsis or complications with chemotherapy and transplant. In terms of OS (Supplementary Figure S2), in the 3-year OS rate no statistically significant differences were found between patients with either hom and hetMNA plus 11q-deleted tumors and patients with homMNA w/o 11q-del tumors (49.2% ±13 versus 53% ±9.5, p = 0.335), although the slope was somewhat delayed in the latter group. When considering the patients with homMNA plus 11q-deleted tumors and the patients suffering homMNA w/o 11q-del tumors the OS were 33.3±13 and 53±9.5 respectively (p = 0.138).

**Table 1:** Clinical characteristics and outcome.

ID	Sex	Age at diagnosis (months)	Stage	Metastases	Pathology	Protocol Treatment	Treatment Response	Relapse	Time to first relapse (months)	Outcome	Follow-up time (months)
1	M	12	1	N	pdNB	INES	CR	Y	14	DOD	15
14	M	41	2	N	nGNB	LNESG1	SurPR	Y	4	DOD	53
5	F	18	3	N	pdNB	N-II-92		N		DOS	1
8	M	33	3	N	uNB	HR-NBL1	PR	Y	28	DOD	58
15	F	9	3	N	pdNB	INES	PR	N		AWD	76
18	M	24	3	N	pdNB	HR-NBL1	PR	N		DTC	7
2	F	41	4	Y(B+BM)	pdNB	HR-NBL1		N		DOD	8
3	M	10	4	Y (B+BM)	uNB	INES	DP	Y	5	DOD	7
4	M	20	4	Y (B+BM)	uNB	NAR-99	DP	Y	12	DOD	12
6	M	19	4	Y (LN)	uNB	NAR-99	VGPR	N		ADF	132
7	F	108	4	Y(B+BM)	pdNB	HR-NBL1	DP	N		DOD	10
9	M	20	4	Y (B+ST)	uNB	HR-NBL1	VGPR	Y	9	DOD	12
10	F	52	4	Y(B+BM)	pdNB	HR-NBL1		N		DTC	3
11	M	14	4	Y (BM)	uNB	NAR-99	CR	Y	9	DOD	10
12	F	40	4	Y (B+BM)	pdNB	HR-NBL1	PR	Y	18	DOD	42
13	M	96	4	Y (B+BM)	pdNB	HR-NBL1	PR	N		AWD	13
16	M	50	4	Y (B+BM)	uNB	HR-NBL1	CR	Y	4	DOD	27
17	F	15	4	Y(B+BM+LN+ST)	pdNB	HR-NBL1	VGPR	N		AWD	48
19	M	41	4	Y (B+BM)	uNB	N-II-92	CR	N		ADF	77

M, male; F, female; N, no; Y, yes; B, bone; BM, bone marrow; LN, lymph nodes; ST, soft tissue; uNB, undifferentiated neuroblastoma; pdNB, poorly differentiated NB; nGNB, nodular ganglioneuroblastoma HR-NBL1, High-Risk Neuroblastoma Study 1;INES,Infants Neuroblastoma European Study, SIOPEN protocols; CR, complete response; VGPR, very good partial response; PR, partial response; DP, disease progression; SurPR, surgical partial resection; DOD, died of disease; DOS, died of sepsis; DTC, died of treatment complication, AWD, alive with disease; ADF, alive disease-free.

The patients have been listed according to the stage of disease. ID was assigned according to genetic aberrations of chromosome 2.

**Table 2:** Histopathological and genetic characteristics of the tumors.

		FISH results				
ID	Pathology	% MNA	% 11q loss (% 11q imbalance)	%1p loss (% 1p imbalance)	% 17q gain	Ploidy
1	pdNB	95	60	25 (10)	60	T
14	nGNB	<5 (het)	60	0	ND	ND
5	pdNB	98	80	90	80	D
8	uNB	95	65	10 (10)	65	T
15	pdNB	5 (het)	50	20	80	T
18	pdNB	90	20	0 (10)	50	D
2	pdNB	95	20 (50 )	ND	ND	TE
3	uNB	95	65	95	65	D
4	uNB	90	35	15 (80)	50	BD -TE
6	uNB	95*	20	31 (64)	50	T
7	pdNB	98	20	98	0	T
9	uNB	95	50	90 (10)	60	T
10	pdNB	90	20	80	50	D
11	uNB	15 (het)	35 (35)	60 (20)	90	T
12	pdNB	30 (het)	50	10 (5)	60	T
13	pdNB	10 (het)	70	ND	ND	D
16	uNB	35 (het)	45	40	ND	T-P
17	pdNB	15 (het)	25	20 (70)	ND	ND
19	uNB	98	50	50	60	ND

uNB, undifferentiated neuroblastoma; pdNB, poorly differentiated NB; nGNB, nodular ganglioneuroblastoma; %, percentage; het, heterogeneous; ND, not done; D, diploid; BD, borderline diploid, T, triploid; TE, tetraploid; P, pentaploid.

All cases presented dmin except case number 5 (* dmin plus HSR).

The patients have been listed according to the stage of disease. ID was assigned according to genetic aberrations of chromosome 2.

Regarding histopathology, according to INPC guidelines eight tumors were undifferentiated NB, ten poorly-differentiated NB and one a nodular ganglioneuroblastoma [23] (Tables 1 and 2). Tumor cell content in all samples was 60% or more.

### Overview of Genetic Markers

In our series of NB analyzed by FISH, MNA was found in 18% of the samples (163 out of 905 tumors). FISH was used to detect aberrations at 1p, 11q and 17q. In this series, 1p deletion was observed in 25% of the tumors (117 out of 477 samples), 11q deletion was detected in 19% of the tumors (92 cases out of 486 samples) and 38% of the samples showed 17q gain (160 out of 420 tumors). Of the 486 cases, 19 primary tumors (4%) presented MNA and 11q loss simultaneously. Table 2 summarizes the FISH and ploidy results for the nineteen primary tumors. Of the nineteen primary tumors, seven showed hetMNA, these cases are described in further detail below. Fifteen cases showed deletion in the 1p36 region. 17q gain was observed in all but one of the fourteen cases studied by FISH. Ploidy was analyzed in sixteen samples. Nine cases were triploid, one of which presented a pentaploid population.

We describe below the limits of the *MYCN* amplicon, the shortest regions of overlap (SROs) in the most recurrent deleted and gained regions, as well as other SCA sizes detected in the original tumors. In addition we provide the genetic intratumoral heterogeneity data.

#### Characterization of genetic markers

**Figure 1:**
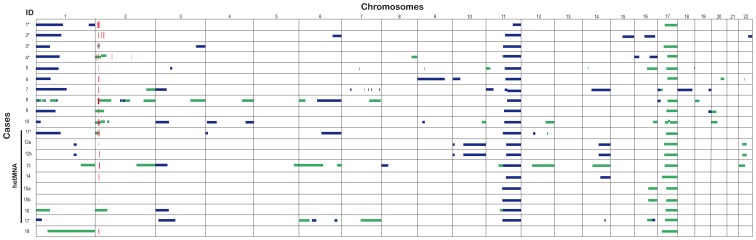
Summary of aSNP data of segmental chromosome alterations. Horizontal lines show segmental loss (blue) and gain (green). Red-vertical lines show amplification. Cases with hetMNA are indicated to the left. Complex MNA are marked by*. The cases have been listed according to chromosome 2.aberrations.

A good concordance was observed between MLPA and aSNP procedures in eighteen out of the nineteen original NB, taking into account the different resolutions of the methods (Figure 1; Table 3 and Table S1). Using aSNP results a high number of SCAs were found in the majority of the cases. The mean SCA of all the cases was 10.7 (range 4–23) (Figure 1 and Table S1). Figure S1 shows a graphic representation of MLPA/aSNP results in case 3 as an example.

**Table 3:** MLPA data.

ID	MLPA results
1	1p−;1q−; MNA; 11q−; +17q
2	1p−; MNA; 11q−
3	1p−; MNA; +3q; 11q−; +17q
4	1p−; MNA; +2p; 11q−; +17q
5	1p−; MNA; 3p−; 9p−; +11p; 11q−; +17q
6	1p−; MNA; 9pq−; 11q−; +17q
7	1p−; MNA;+2q; 3p−; 11q−; 14q−;17p−; +17p
8	1p−;+1p; MNA; +2p; +2q; +3q; +4q; +7q; 11q−; 17p−;+17q
9	1p−; MNA; +2p; 11q−; +17q
10	1p−; MNA; +2p; 3p−; 4p−; 4q−; 9p−; 11q−;+17q
11	1p−; MNA; +2p; 6q−; 11q−; +12q; +17q
12a	*MYCN* gain; 11q−; 14q−; +17q;
12b	MNA; +11p, 11q−; 14q−; +17q
13	+1q; MNA; +2q; 3p-; +11p; 11q−; +12q; +14q; +17q
14	MNA; 11q−; 14q-; +17pq
15a	11q−; +17q
15b	*MYCN* gain; 11q−; +17q
16	+1p; +2p; 3p−; 11q−; +17q
17	1p−; 3p−; +7q; 11q−; 14q−+17q
18	+1pq; MNA; +17q

INRG Biology Committee definitions [11]. *MYCN* amplification (MNA), up to 4-fold excess of signal numbers of the chromosomal region of interest compared with the reference signals; Gain (+), unbalanced ratio (high signal excess) between the signals of a gene and all other probes located on the same chromosome; Loss (−), unbalanced ratio (low signal excess) between the signals of the chromosomal region of interest (at least two adjacent probes) and the reference signals (at least two) of the chromosomal region of interest.

The cases have been listed according to chromosome 2 aberrations using pangenomic techniques.

**Figure 2:**
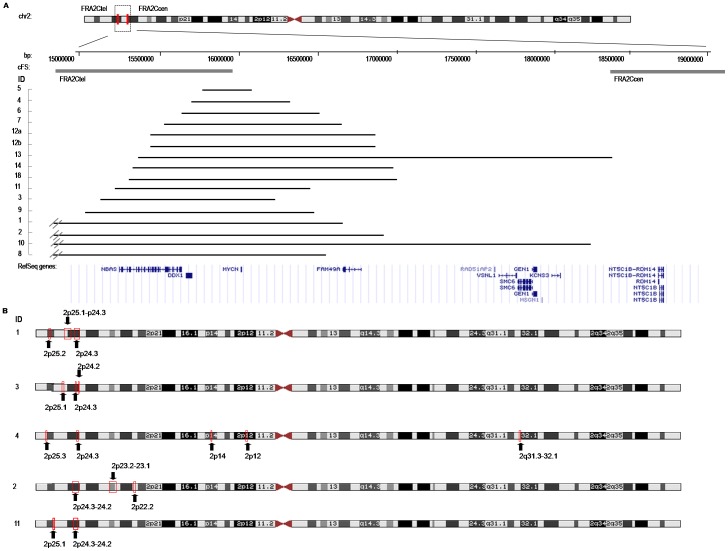
Schematic representation of the amplicons in chromosome 2. All data are illustrated according to the NCBI build Hg19. (A) Genes included in the *MYCN* amplicon. The crossed lines in cases 1, 7, 9 and 10 indicate that the breakpoint mapped more distal in chromosome 2 than the area represented. (B) Boxes and arrows indicate position of the different amplicons in the cases with complex amplification. The cases have been listed according to chromosome 2 aberrations.

**Figure 3:**
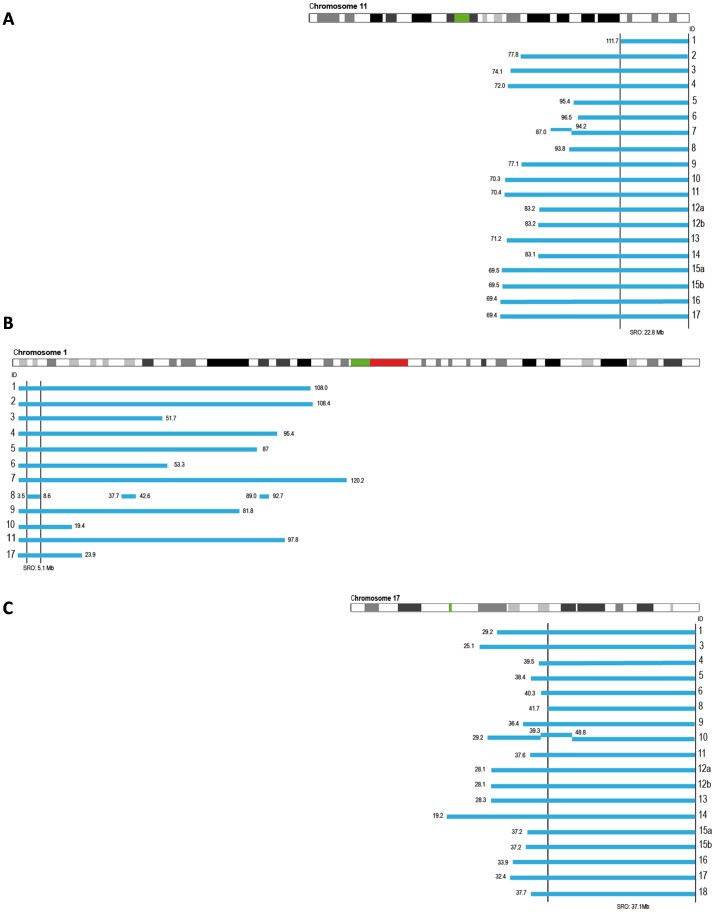
Graphic representation of chromosomes 11, 1 and 17. Bars illustrate the deleted/gain region. The positions of the breakpoints are indicated in megabases. (A) Deletions of chromosome 11q. SRO: 1117.7 to qter (22.8 Mb). (B) Deletions of chromosome 1p. SRO: 3.5 to 8.6 Mb (5.1 Mb). (C) Gain of chromosome 17q. SRO: 41.7 to qter (37.1 Mb). The cases have been listed according to chromosome 2 aberrations.

Fifteen cases showed MNA by multilocus/pangenomic techniques. A great variability in the total region size of the *MYCN* amplicon (from 600 kb to 6.3 Mb) and amplicon gene composition between the tumors was found in both homMNA and hetMNA (Figure 1 and Table S1). Two different MNA patterns were found: simple amplification with one continuous region amplified (ten cases; three of which were hetMNA) and complex rearrangements with several discontinuous amplification regions (five cases; see Figure 1 cases indicated with *, and Figure 2A, 2B). Five out of fifteen cases showed both MNA and 2p gain. Three groups can be formed according to the genes included in the amplified *MYCN* region: only *MYCN* gene (case 5), *DDX1-MYCN* gene (cases 4 and 6), and *DDX1*-*MYCN* with *NBAS* (entirety or in part) genes (twelve samples). In the first two groups, most telomeric breakpoints of the *MYCN* amplicon were located slightly proximal to the fragile site *FRA2C*tel at 2p24.3. In the third group the distal breakpoints were mapped within *FRA2C*tel, a fragile site located at 2p24.2 (eight cases) or located more distally to this critical site (four cases) (Figure 2A). Case 2 presented an amplified peak at p23.2-23.1 containing the whole gene *ALK* within. Case number 4 showed one small amplification (2q 31.3–32.1) concomitantly with four amplification peaks in 2p region (Figure 2B). MLPA/aSNP verified deletion of the long arm of chromosome 11 in seventeen cases. The consensus region of 11q loss was from 111.7 to 134.5 Mb (q23-qter), SRO being 22.8 Mb. The mean size of 11q loss was 53.7 (range 22.8–64.9 Mb). The deleted region was uninterrupted in all cases except in case 7 (two breakpoints were displayed) (Figure 3A).

Twelve cases showed 1p loss using MLPA/aSNP techniques confirming the FISH results (Figure 3B). The consensus region of 1p loss ranged from 3.5 to 8.6 Mb, with 5.1 Mb of SRO. The mean size of 1p deletion was 65.3 Mb. Continuous deletion along 1p36 region was present in eleven cases with a larger deleted region in ten cases (deletion size >20 Mb) (Figure 3B). Case 8 presented complex chromosome 1 rearrangements with up to 7 SCAs in 1p-arm. Gain of 1q was observed in case 13. Loss of 1q was detected as terminal (case 1) and interstitial (case 12) (Figure 1 and Table S1). Case 18 presented a large gain region covering both the p and the q arms.

Sixteen cases presented 17q gain. In addition, case 8 showed 17p loss. In case 7 17p loss was observed (pter-14.4 Mb) by MLPA/aSNP without associated 17q gain (Figure 1 and Table S1). The mean 17q gain was 45.3 Mb. The consensus gained segment was rather large, extending from 47.1 Mb to qter (37.1 Mb) (Figure 3C).

Deletion in 3p was observed in 33% of cases (6/18). In cases 5 and 17, interstitial deletions were observed, while the rest of the cases showed common terminal 3p deletions. In case 10, deletions in both arms of chromosome 4 were seen. A small 4p deletion was observed in case 11 (size 10 Mb). Cases 5 and 10 showed an interstitial 9p deletion of 0.4 and 12.2 Mb respectively. A hemizygotic deletion of *CDKN2A/B* and *MTAP* genes, located in 9p21.3, was observed in case 5. Other chromosome rearrangements were found in several cases (Figure 1 and Table S1).

An aSNP allele analysis revealed several large copy neutral loss of heterozygosity (CN-LOH) in different chromosomes. CN-LOH was detected in four cases (1, 5, 10 and 12), although without satisfying the requirement for matched constitutional DNA. In case 1, eight chromosome regions presented CN-LOH (1q, 2p, 5q, 7p, 9p, 9q, 10p and 17p). Cases 5, 10 and 12 presented CN-LOH in chromosome 9p, 11p and 18q respectively.

#### Intratumoral heterogeneity

SCAs diagnosed by presence or absence of specific DNA sequences using fluorescent probes can be complex, with a miscellany of clones, some of which can remain hidden when using MLPA/aSNP approaches, resulting in different genetic states associated with the *MYCN* gene, and 11q, 1p and/or 17q chromosome regions within a single tumor. Intratumoral heterogeneity was present in cases 11 to 18 (42%) and considered as heterogeneous SCA (hetSCA) cases. Total percentages of neuroblasts with SCAs detected by FISH in the tumor cohort are presented in Table 2. Details of the different frequencies of clusters with SCA, NCA as well as disomic cells seen in the hetSCA tumors are presented in Table 4. Co-existence of cells with SCA along with NCA cells in the hetSCA tumors was often present for all analyzed markers. Co-existence of hetMNA and het11q-del was present in the 31.5% of the cases (six out of nineteen samples) analyzed by FISH. However, cases 14 and 15 showed co-existence of MNA neuroblastic cells and disomic tumor cells confirmed by histopathological study (data not shown). Multilocus/pangenomic data are shown in Figure 1, Table 3 and Table S1. MNA was detected in fourteen out of eighteen (77.8%) of the cases with these approaches. Regarding detection of hetMNA status (cases 11 to 17), multilocus/pangenomic techniques were accurate in three cases. Four out of seven hetMNA cases showed MNA in some of the pieces analyzed by MLPA/aSNP (cases 11 to 14). Case number 15 presented *MYCN* gain in one of the two pieces studied. A 2p gain of a large region (56 Mb) was detected in case 16. No SCA involving 2p was found in case 17. Three hetMNA cases (samples 12, 15, 16) were diagnosed as having heterogeneous 1p deletion. The percentage of tumor cells with this alteration by FISH ranged from 10 to 40%. Heterogeneity of 11q deletion (het11q-del) was found in case 18 (5.5%), where this aberration was revealed by FISH in only 20% of tumor cells, but was not shown by multigenomic analysis.

**Table 4:** FISH results in the heterogeneous cases (hetMNA and het11q-del).

ID	MYCN	11q	1p	17q
11	**5∶35 15%;** 5∶5 80%; 2∶2 5%	**2∶1 35%;** **3∶2 35%;** 3∶3 15%; 2∶2 15%	**(4∶1, 5∶1, 6∶1) 60%;** (4∶2,5∶2,6∶2,4∶3,5∶3,6∶3) 20%; 2∶2 20%	**2∶4 30%; 3∶5 30%; 3∶4 30%;** 2∶2 10%
12	**2∶50–100 dmins 10%, 4∶50–100 dmins 20%;** 4∶4 40%; 2∶2 30%	**2∶1 25%; 3∶1 25%;** 3∶3 10%; 2∶2 40%	**2∶1 10%; 4∶2 5%;** 4∶4 35%; 2∶2 50%	**2∶3 50%; 2∶4 15%; 2∶5 15%;** 2∶2 20%
13	**2:>100 dmins 10%;** 2∶3 40%; 4∶4 10%; 2∶2 40%	**3∶1 70%;** 3∶3 10%; 4∶4 5%; 2∶2 15%	ND	ND
14	**2∶20 dmins <5%;** 2∶2 95%	**2∶1 60%;** 2∶2 40%	3∶3 10%; 4∶4 15%; 2∶2 75%	ND
15	**2:>100 dmins 5%;** 2∶2 95%	**2∶1 25%; 3∶1 25%;** 3∶3 10%; 2∶2 40%,	**2∶1 20%;** 2∶2 80%	**2∶3 50%; 2∶4 15%; 2∶5 15%;** 2∶2 20%
16	**(2∶10,2∶12) 35%;** (4∶6,2∶3,2∶6) 30%; 2∶2 35%	**2∶1 15%;** **3∶1 30%;** 3∶3 15%; 2∶2 30%	**2∶1 40%;** 2∶2 60%	ND
17	**4:>50 dmins 10%; 2:>50 dmins 5%;** 4∶4 60%; 8∶8 5%; 2∶2 10%	**3∶1 25%;** 3∶3 15%; 2∶2 60%	**4∶1 10%; 2∶1 10%;** 4∶2 70%; 2∶2 10%	ND
18	**3:>100 dmins 20%, 2:>100 dmins 20%, 4:>100 dmins 50%;** 2∶2 10%	**2∶1 20%;** 2∶2 80%	**2∶3 10%;** 4∶4 10%; 2∶2 80%	**2∶3 50%;** 2∶2 50%

Disomic cells (ratio 2∶2) and numeric alterations (ratios 3∶3; 4∶4…), balanced ratio between the signal numbers of chromosomal region of interest and the reference signals on the opposite arm of the chromosome; Cells with gain (ie ratios 2∶4; 3∶4…), signal numbers of the chromosomal region of interest exceed up to 4-fold the number of reference signals; Cells with imbalance (ie ratios 3∶2; 4∶3…), imbalance ratio between the signal numbers of chromosomal region of interest and the reference signals with more than 1 signals of chromosomal region of interest; Cells with deletion (ie. ratios 2∶1; 3∶1 4∶1…), unbalanced ratio between the signal numbers of the chromosomal region of interest and the reference signals with only 1 signal of the chromosomal region of interest; hetMNA, occurrence of clusters or as single cells with amplification (at least five cells per slide) surrounded by non-amplified tumor cells.

ND, not done.

The cases have been listed according to chromosome 2 aberrations using pangenomic techniques.

## Discussion

NB tumors show a diverse behavior, and an algorithm of clinical, histopathological, and genetic factors has stratified the risk and delineated therapeutic decisions [6], [13], [20]. Nevertheless, NB demands improved characterization and a better understanding of how tumor biology drives specific clinical behavior, especially for those patients identified as having a high-risk phenotype [6]. The present study analyzes, for the first time, the genetic characteristics in the largest cohort to date of unusual neuroblastic tumors carrying MNA plus 11q deletion, using the aSNP technique in addition to FISH and MLPA techniques to obtain high resolution detection and mapping of numerical and structural genomic changes in these tumors.

MNA and allelic loss of 11q with single copy of *MYCN* are associated with advanced stage NB, and both are independent prognostic indicators for clinically high-risk patients [19], [22], [29]. Despite this, hetMNA status is not prognostically interpretable worldwide, and the effect of 11q loss on NB biology is not sufficiently clear [15], [21]. Several hypotheses have already been published related to the aggressiveness of hetMNA clone(s), varying from a ‘premalignant’ status lacking malignant properties to an invasiveness and metastatic potential comparable to homMNA tumors [12], [15], [16], [30]. Of the surviving patients described in this study, 60% correspond to cases with hetMNA; however, no significant prognostic differences in survival were found between patients with homMNA plus 11q-deleted tumors and the patients with hetMNA plus 11q deletion tumors. The homMNA w/o 11q-del and hom and hetMNA plus 11q-deleted groups were similar in terms of overall survival in the analyzed cohort. Previous studies showed a worse outcome in the group of MNA tumors plus 11q loss than in patients with MNA alone [22]. These differences could be due both to the presence of hetMNA tumors in our cohort and the low number of cases with homMNA. In fact, the survival analysis excluding hetMNA tumors showed a trend toward statistical differences in OS rates, suggesting a less aggressive nature for tumors with hetMNA than tumors with homMNA. The SIOPEN is now studying the dilemma created by these unusual cases. Studies of a large number of samples have shown differences in age at diagnosis between the MNA group and 11q-deleted group [22], [29]. In a recent study, the median age at diagnosis in MNA cases was lower than in the 11q loss group [19]. In the cohort of infrequent tumors herein presented, the median age at diagnosis was comparable to that of the group with MNA. A significantly higher frequency of chromosomal breaks has been reported in NB with 11q deletion than in MNA NB cases (12 versus 4) [19]. The cases analyzed here shared a high number of SCAs (mean 10.7; median 10) with the 11q-deleted group.

Both simple and complex types of MNA amplicon organization have been described using the aSNP technique in NB [31]–[33]. The copy number of the *DDX1* gene, located in the close vicinity of *MYCN*, has controversial prognostic implication and was amplified in all but one of the cases analysed [32], [34]–[38]. *NABS,* overexpressed via *MYCN* co-amplification, may perturb the quantitative balance of complexes involved in cell-cycle-related events and membrane trafficking [36]. This gene was also frequently co-amplified with *MYCN* in our series. Co-amplification of the entire *ALK* gene is reported to occur rarely, it is observed only in patients with poor outcome, and is not a statistically significant independent marker for survival [39]. In this series, only one case presented *ALK* amplification. Recently, Blumrich et al. [40] suggested that *MYCN* amplicons arise from extra replication rounds of secondary DNA structures accumulated at *FRA2C*tel and/or *FRA2C*cen. Genomic location of the *FRA2C* subregion has been described, being able to accurately define the borders of the region using BAC clones. The complex genomic rearrangements in the boundaries of the common fragile sites (cFS) has led to the proposal of a dual role for the cFS in the generation of gross chromosomal rearrangements, either after DNA breakage or by inducing extra replication rounds [40]. Our results are consistent with this study and revealed that most amplicon borders were clustered within the *FRAC2*tel, and that most of the proximal amplicon borders were located in the region spanning 15.5 and 16.6 Mb on 2p. An association between MNA and 2p gain has been described previously [31], [32], [41]. In our study, 33% of the tumors with MNA presented 2p gain using MLPA/aSNP but, and in agreement with other studies, no relevant clinical differences in overall survival were observed between these samples and the samples without 2p gain (data not shown) [41].

In 11q-deleted cases, different candidate suppressor genes have been proposed [17]–[19]. The loss of one copy of the *H2AFX* gene (11q23.2–q23.3) has been described as the factor responsible for the genetic instability in the 11q-deleted NB due to its role in double-strand break repair and cancer susceptibility [19]. This hypothesis implies accepting that the 11q deletion event must occur early in tumorigenesis, despite the relatively old age of patients seen in some studies. However, studies of gene expression profiles suggest that 11q loss is acquired in unfavorable NB as a result of selective pressure and confers specific properties to the tumor, while MNA would be an earlier event [21], [42]. All the patients of the infrequent group described in this study presented tumors with neuroblasts with losses of gene *H2AFX* and with homMNA or hetMNA along with genetic instability and young age at diagnosis. We can hypothesize that a younger age can be related to loss of gene *H2AFX* and hetMNA as early events in tumorigenesis.

Simultaneous genomic imbalances can strengthen one another or act in combination, which may affect other perfectly-balanced genomic regions [43]. Deletions of chromosome arm 1p have been well characterized, and different SROs have previously been reported in NB, occurring in approximately 30–35% of primary NB and being highly correlated with MNA [6], [19]. There is evidence that one or more tumor suppressor genes involved in NB initiation and/or progression are localized to this region [44]. Tumors with MNA have 1p deletions extending proximal to 1p36, while *MYCN* non-amplified tumors, including cases with 11q loss, more often have small terminal deletions of 1p36 [19], [31]. Caren et al. [31] located an interstitial SRO between 17.2 Mb and 37 Mb for the MNA tumors and an SRO from 0 to 10.4 Mb for the second group. A larger study established the SRO at 5.3 Mb and 6.1 Mb [19]. Our SRO apparently matches with the SRO previously reported in tumors without MNA. Recently, a recurrent 17.9 Mb terminal deletion on distal chromosome 1q has been reported in association with high-risk NB with 11q deletion without MNA [45]. In our study, only one case presented a similar terminal deletion (19 Mb, q42.13-qter). Two possible candidate tumor suppressor genes located within the deleted segment are *FH* (fumarate hydratase) and *EGLN1* (EGL nine homolog 1), implicated in other types of tumors [45]. Furthermore, different experiments suggested that chromosome 1q gain, seen in one case of this cohort, is associated with poor outcome in NB patients [1].

It is not surprising that the majority of the cases presented gain of 17q. Gain of genetic material on the long arm of chromosome 17 is very common in aggressive NB, irrespective of presence or absence of MNA. 17q gain and the breakpoint position on 17q may influence tumor behavior [46]. In our study, the MLPA/aSNP profile was confirmatory of a single 17q breakpoint (leading to a single region of gain) in all cases but one. The resulting copy number gain for 17q invariably implicated a large segment encompassing at least the distal part from approximately 41.7 Mb to qter. These results are in keeping with the results obtained in other high resolution studies where various oncogenes have been proposed [46]. Critical genes sensitive to a gene dosage effect contributing to NB pathogenesis could be located in the SRO (37.1 Mb) of our study. In addition, three of our cases presented 17p loss, an alteration that has been related with chemoresistant NB [47].

Loss of chromosome 3p is a frequent event in NB, occurring preferentially in tumors that exhibit loss of 11q [22]. In this study, samples showing loss of 3p had patterns equivalent to those previously described. Critical regions of minimal 3p loss contain multiple candidate tumor suppressor genes, which may contribute to NB pathogenesis [31], [48]. Other typical SCA with unclear prognostic significance in NB, such as deletion of 4p or 9p, were detected in the tumors analyzed [1]. Although, CN-LOH and homozygous deletions are rare events in NB, some cases have been reported and were present in the tumor cohort analyzed. Their clinical impact is unknown [31], [49]. As no matched constitutional DNA was investigated for any of our samples, no conclusion about acquired or inherited CN-LOH can be drawn.

High intra-sample genomic heterogeneity, characteristic of some NB, as well as stromal DNA contamination may explain discordant results between the techniques [8], [50]. When using multilocus/pangenomic techniques to analyse tumor samples, intratumoral genetic hetereogeneity, complex patterns of CNA, LOH events and polyploidy as well as normal DNA contamination effects must be considered. Current algorithms, although robust computational methods, try to correct for the presence of normal cells allowing at the same time the detection of CNA occurring in only a subset of cells. Up to now it has often been proved to be sub-optimal [27], [51]. Especially in NB, a single statistical framework able by itself to overcome the complex genetic heterogeneity should be used to improve the analysis of tumor pangenomic data. In this regard, FISH has a higher sensitivity because it detects the gene or region copy number at the single cell level and allows the correlation of morphologic details, while at the same time it is not affected by stromal contamination. As we present here, hetSCAs were not observed easily by MLPA/aSNP. Thus far, hetSCA status in NB can remain masked and is therefore difficult to diagnose, creating the need for additional information for diagnosis, prognostication and therapeutic decision making [12], .

In summary, we have made a detailed study of a subgroup of neuroblastoma with both MNA and 11q deletion. We found that this group of tumors has approximately the same high frequency of aberrations as earlier found for 11q deleted tumors, albeit not higher. These tumors represent a valuable model of genetic instability, revealing intratumoral heterogeneity in NB. An in-depth investigation of the genome of these same cases should be made in future studies, using new generation sequencing techniques to identify candidate genes associated with tumor progression and potential target genes for drug therapy in this subgroup of high-risk patients.

## Supporting Information

Figure S1 **Graphic representation of MLPA/aSNP results in case number 3.** Chromosomes with segmental aberrations are indicated by an arrowhead. (A) Graphic results obtained using NB probemixes P251, P252 and P253. The thresholds for loss and gain detection were set at 0.75 and 1.25, respectively. Normal values are showed in yellow bars, gains and amplification in green bars and losses in red bars. (B) Whole genomic profile of the aSNP and single view of chromosome 2p arm. The figure displays the sequential amplicons detected.
(TIF)

Figure S2 **Kaplan-Meier overall survival curves.** (A) Patients with hom and hetMNA plus deleted tumors (n = 16) and homMNA w/o 11q-del tumors (n = 28); 3-year overall survival: 49.2% ±13 versus 53% ±9.5, p = 0.335. (B) Patients with homMNA plus 11q-deleted tumors (n = 9) and homMNA w/o 11q-del tumors (n = 28); 3-year overall survival 33.3±13 versus 53±9.5, p = 0.138.
(TIF)

Table S1 **Summary of aSNP data of segmental chromosome alterations.** The table shows the sizes of different segments with amplification (red), gain (green) or loss (blue). Complex MNA are marked with an asterisk * in the first row ‘ID
(TIF)
