# Supplementary figures and images for: Genetic Instability and Intratumoral Heterogeneity in Neuroblastoma with MYCN Amplification Plus 11q Deletion
Source: PLoS One. 2013 Jan 14;8(1):e53740. doi: 10.1371/journal.pone.0053740 (PMC3544899; doi:10.1371/journal.pone.0053740)

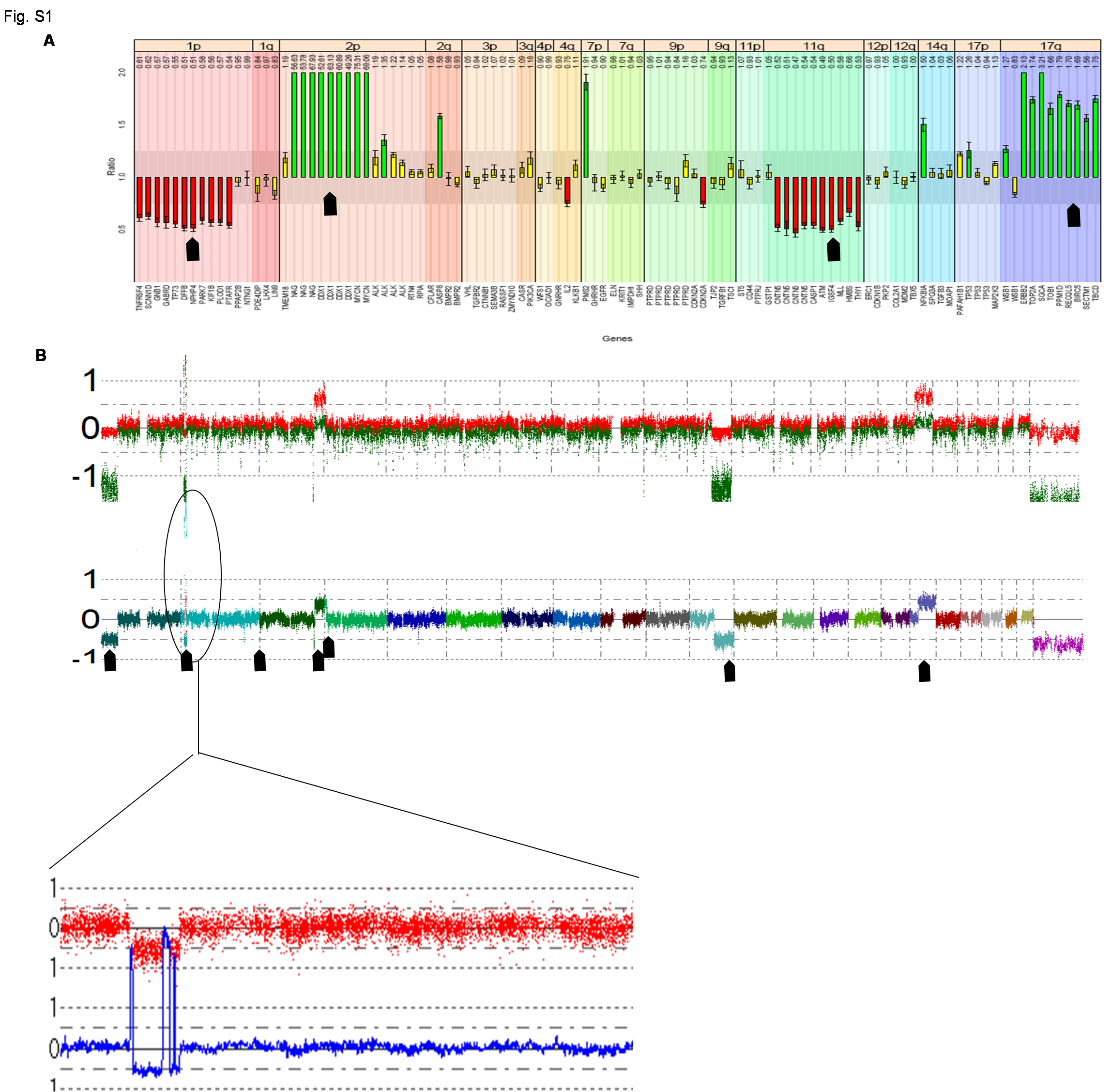

Supplement: Figure S1 — Graphic representation of MLPA/aSNP results in case number 3. Chromosomes with segmental aberrations are indicated by an arrowhead. (A) Graphic results obtained using NB probemixes P251, P252 and P253. The thresholds for loss and gain detection were set at 0.75 and 1.25, respectively. Normal values are showed in yellow bars, gains and amplification in green bars and losses in red bars. (B) Whole genomic profile of the aSNP and single view of chromosome 2p arm. The figure displays the sequential amplicons detected. (TIF) [file pone.0053740.s001.tif]

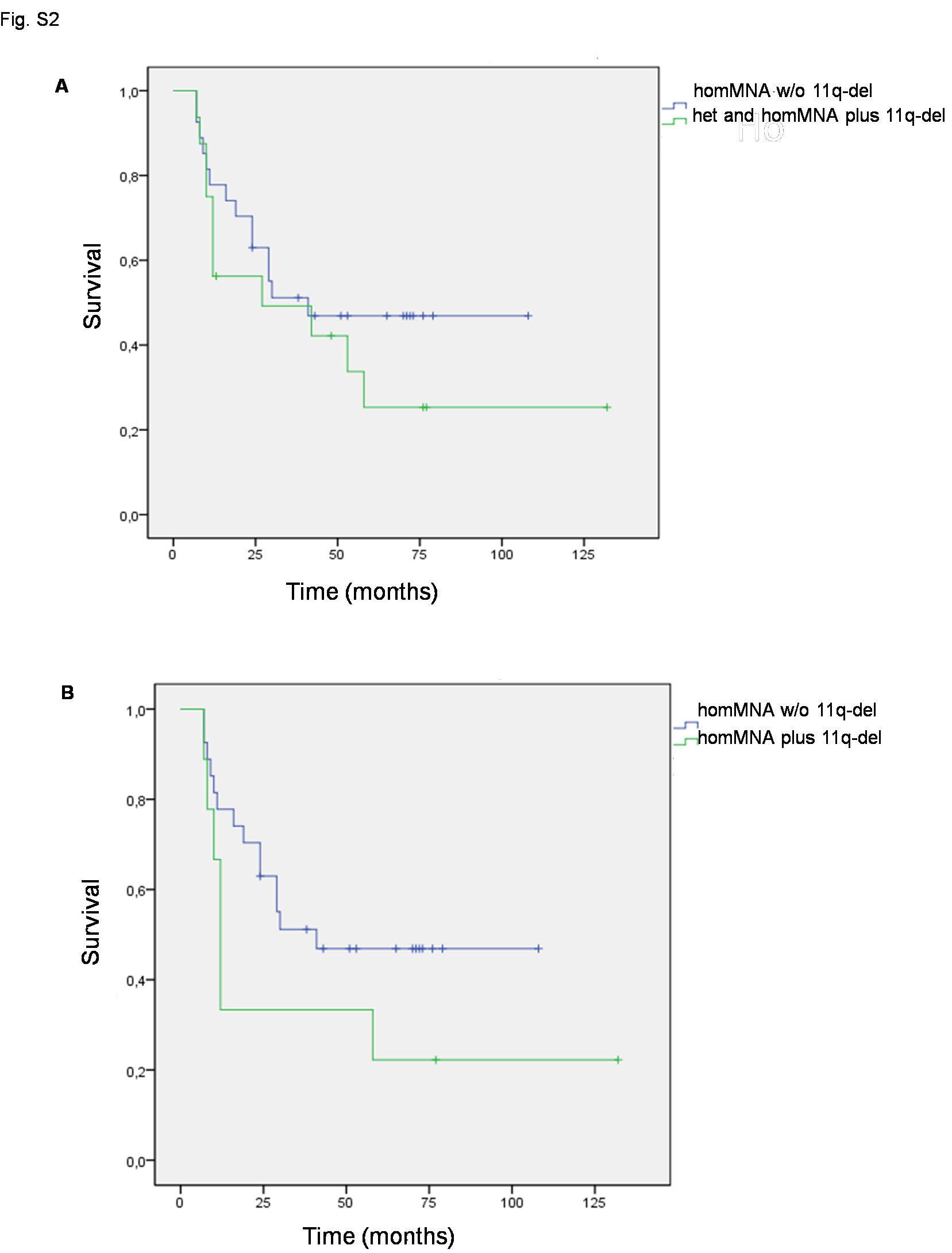

Supplement: Figure S2 — Kaplan-Meier overall survival curves. (A) Patients with hom and hetMNA plus deleted tumors (n = 16) and homMNA w/o 11q-del tumors (n = 28); 3-year overall survival: 49.2% ±13 versus 53% ±9.5, p = 0.335. (B) Patients with homMNA plus 11q-deleted tumors (n = 9) and homMNA w/o 11q-del tumors (n = 28); 3-year overall survival 33.3±13 versus 53±9.5, p = 0.138. (TIF) [file pone.0053740.s002.tif]

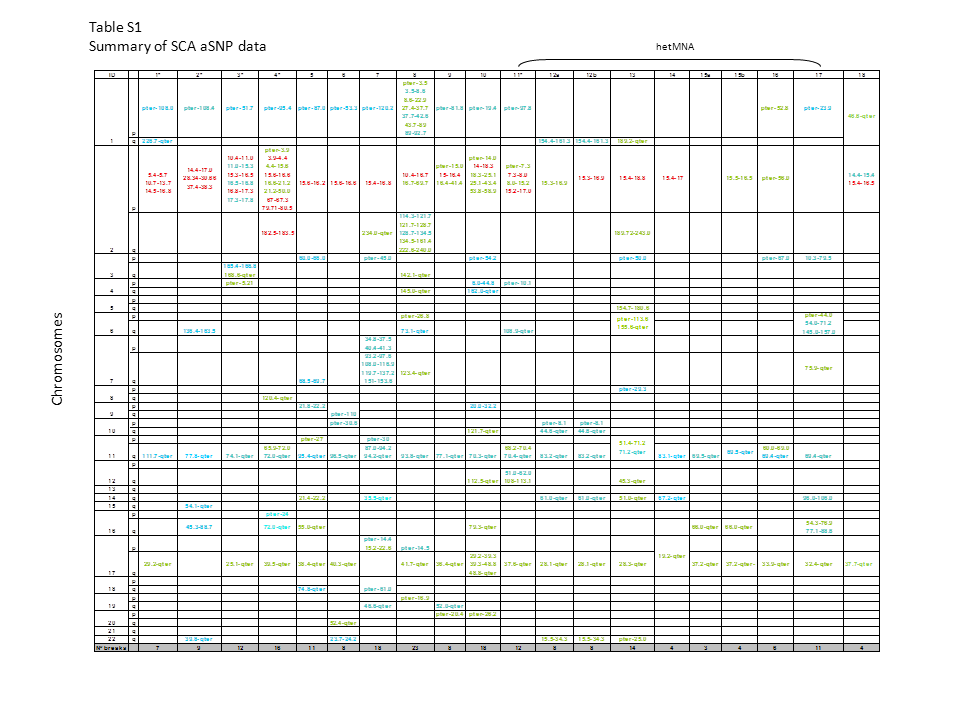

Supplement: Table S1 — Summary of aSNP data of segmental chromosome alterations. The table shows the sizes of different segments with amplification (red), gain (green) or loss (blue). Complex MNA are marked with an asterisk * in the first row ‘ID (TIF) [file pone.0053740.s003.tif]
